# Facial morphology analysis of Caucasian Brazilian adult women using stereophotogrammetry

**DOI:** 10.1590/1807-3107bor-2024.vol38.0105

**Published:** 2024-11-08

**Authors:** Maria Gabriela Robles Mengoa, Amanda Aparecida Maia Neves Garcia, Karolyn Sales Fioravanti, Karin Hermana Neppelenbroek, Thaís Marchini Oliveira, Chiarella Sforza, Simone Soares

**Affiliations:** (a) Universidade de São Paulo – USP, Bauru School of Dentistry, Department of Prosthodontics and Periodontics, Bauru, SP, Brazil.; (b) Universidade de São Paulo – USP, Bauru School of Dentistry, Department of Pediatric Dentistry, Orthodontics and Public Health, Bauru, SP, Brazil.; (c) Università degli studi di Milano, Department of Biomedical Sciences for Health Milan, Italy.

**Keywords:** Photogrammetry, Aging, Face, Female, Anthropometry

## Abstract

This cross-sectional observational study aimed to evaluate and compare facial metrics in women aged 20–65 years using a three-dimensional (3D) stereophotogrammetry system and to establish standardized values for facial metric variations in different age subgroups. This study included 84 Caucasian women divided into two groups based on their age: group 1 (G1) included women aged 20–40 years and group 2 (G2) included women aged 41–65 years. Twenty-one morphometric points on the face were identified, and the facial images were captured using a 3D stereophotogrammetry system, Twenty-three linear measures and 12 angular measures were evaluated, revealing statistically significant differences in 11 linear and 5 angular measures between the groups. In the G2 group, nasal and mouth width, lip philtrum height, Tragus-Nasion and Tragus-Pronasale lengths were increased, along with increased nasofrontal angle, decreased palpebral fissure inclination, and lip vermilion angles. However, palpebral fissure width and height, binocular width, and lip vermilion height were reduced. The aging process in women causes substantial changes in facial features, particularly in the middle and lower thirds of the face. Conversely, no major changes were observed in the upper third of the face. Our study findings provide potential insights for clinicians in developing facial rejuvenation procedures as well as for forensic purposes and surgical planning. The standardized facial metrics values in different age subgroups can guide clinicians in determining appropriate treatment plans for patients seeking facial rejuvenation.

## Introduction

The human face undergoes various physical changes throughout life.^1,2^ Although there is a general aging pattern in each anatomical structure,^3^ investigating the onset and progression of human facial aging in different populations is crucial because of individual, ethnic, and racial variations. Anthropometric techniques have been developed to compare facial differences among individuals, races, and ethnic groups.^4^ Facial anthropometry is the study of the dimensions and measurements of the human face.^5^ Various methods have been developed for performing anthropometric measurements. The traditional method relies on instruments and is time-consuming, unsuitable for children, and examiner-biased.^6–8^ Two-dimensional (2D) photographs exhibit limitations with respect to lighting conditions, variations in camera angle, and the absence of depth cues within facial features.^7,8^ Technological developments have replaced 2D photographs for facial analysis with three-dimensional (3D) methods, such as stereophotogrammetry. Stereophotogrammetry is an advanced imaging technology that allows for more accurate diagnoses.^5^ Moreover, this technology is non-invasive, quick, free of ionizing radiation, and is a safe alternative for evaluating facial morphology, and creates a comprehensive 3D database of the patient's facial soft tissues.^9,10^ Recent advancements in stereophotogrammetry have led to its increased use in both educational research and clinical applications.^10^ Therefore, stereophotogrammetry is a reliable alternative for capturing and analyzing facial changes over time. Establishing standardized values for the proportions, distances, and facial angles in different age subgroups is crucial for distinguishing and comparing facial patterns within populations, which can serve as a guide for orofacial surgical procedures and aesthetic treatments. This standardization will also aid in evaluating and analyzing treatment outcomes.^11,12^ Currently, 3D analysis data on facial morphological variations in the Brazilian population is scarce.^13^ Therefore, this study aimed to evaluate and compare facial metrics among Brazilian Caucasian women of different ages to provide insights into how facial morphology changes with aging.

## Methods

### Ethical approval and informed consent

This study was approved by the ethics committee of the institution where the study was conducted (CAEE: 22075219.6.0000.5417, under protocol number 3.718.125), and was conducted in accordance with the ethical standards of the Declaration of Helsinki. All participants signed an informed consent form before inclusion in the study.

### Study design

This cross-sectional observational study included 84 Caucasian Brazilian women aged 20–65 years.

### Study sample

The sample size calculation was based on a previous study^14^, considering a minimum relevant difference of at least 2.3 mm in soft tissue change (standard deviation, 2.97 mm). With a significance level of 0.05 and a test power of 0.80, the required sample size was calculated to be a minimum of 28 patients per group.

All the included 84 participants (n = 84) had occlusal stability and were undergoing routine dental care at the Bauru Dental School, University of São Paulo. Based on their age, the participants were divided into the following groups: Group 1 (G1), included 37 women aged 20–40 years and Group 2 (G2), included 47 women aged 41–65 years.

Individuals with injuries, previous trauma, and/or tumors in the craniofacial region capable of altering facial anatomy, occlusal instability, congenital or acquired deformities, previous facial surgery and aesthetic treatments, and peripheral and/or central neurological disorders were excluded.

### Anthropometric landmarks

**Figure 1:**
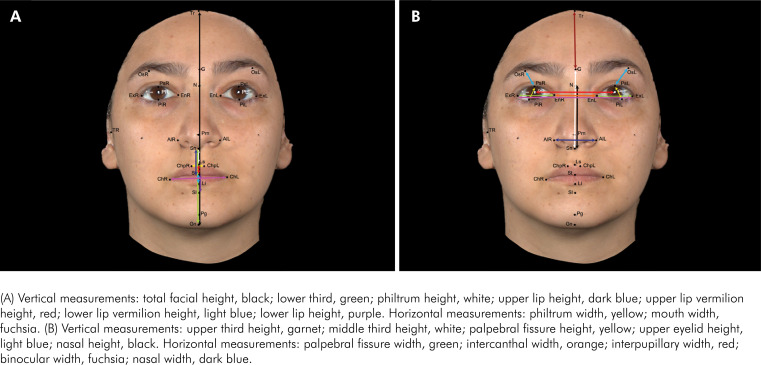
Linear measurements (Frontal view): Images obtained using Vectra Analysis Module Software (VAM elaboration, Canfield Scientific Inc.).

Twenty-one anthropometric landmarks described by FARKAS, 1994^15^ (Table 1 and Figure 1) were included in this study. Of these, 10 landmarks were marked directly on the participant's face by an examiner using an eyeliner (Make B., O Boticário, Brazil). These landmarks were selected and identified by two previously calibrated examiners because of their ease of location through palpation and visual inspection. These landmarks corresponded to: Trichion (Tr), Glabella (G), Nasion (N), Pronasale (Pr), Subnasale (Sn), Labiale superius (Ls), Cph (Crista philtri), Labiale inferius (Li), Gnathion (Gn), and Tragion (Tr).

**Table 1:** Abbreviations and definitions of the selected anthropometric landmarks used in this study.

Abbreviation	Landmarks	Definition
Tr	Trichion	Midpoint of the hairline in the midaxis of the face.
G	Glabella	Midpoint between the center of the eyebrows
Os	Orbitale superius	Highest point on the lower border of the eyebrow
Ps	Palpebrale Superius	Highest point on the free margin of each upper eyelid
P	Pupil center	Midpoint of the iris
Pi	Palpebrale Inferius	Lowest point on the free margin of each lower eyelid
En	Endocanthion	Most medial point of each palpebral fissure
Ex	Exocanthion	Most lateral point of each palpebral fissure
N	Nasion	Point in the middle of both the nasal root and the nasofrontal suture
Prn	Pronasale	The most anterior midpoint of the nose tip
Sn	Subnasale	Central junction of the nasal septum and upper lip
Al	Alare	Lateral point of each nasal alare
T	Tragion	Highest point of each tragus
Sl	Sublabiale	The most posterior point of the mentolabial soft tissue contour
Pg	Pogonion	Most anterior point of the chin
Ls	Labiale superius	Midpoint of the upper vermillion line
Cph	Crista philtri	Point at the intersection of the vermilion line and elevated margin of the philtrum
Ch	Cheilion	Most lateral point of the horizontal lip fissure when the mouth is closed
St	Stomion	Midpoint of the horizontal cleft lip when the mouth is closed
Li	Labiale inferius	Midpoint of vermilion of the lower vermillion line
Gn	Menton or Gnathion	Lowest midpoint of the chin

The identification and reproducibility of the landmarks and marker positioning have been tested and found reliable^16^.

### Facial scanning (stereophotogrammetry)

The Vectra H1 3D stereophotogrammetry equipment (Canfield Scientific, Inc., Fairfield, USA) and VAM elaboration software (Canfield Scientific, Inc.) used in this study have been validated to perform facial analysis.^10,17^ The portable camera system VECTRA H1 is reliable in assessing linear, angular, and area measurements,^10^ making this method accurate and reliable for most clinical and research applications.^17^

The participants were instructed to sit upright in a chair with their heads positioned in a natural position,^18^ and their eyes focused on a stationary point while relaxing their facial muscles and touching their lips lightly. To avoid interference, disposable caps were used to cover the hair, and metallic objects, such as earrings and piercings, were removed to prevent reflections from flash light.

The camera was positioned at a height of 45° to the right of the participant, and green laser dots were used as alignment guides. Three images were taken from different positions: the right side, the front, and the left side of the participant. The capture took approximately 20–30 s, and the images were transferred to a computer for processing. The VAM Module Elaboration software was used to automatically combine these images to create a 3D facial model, which requires calibration to ensure the accuracy of the final facial model.^17^

### Digital anthropometric landmarks

The 3D-reconstructed images of the participants were digitally marked with landmarks by a trained operator using the calibrated method. The first step involves centering and zooming the image to visualize the 10 landmarks that were previously manually marked. These landmarks were identified and digitally marked by placing points at their centers on the 3D images. Next, the remaining 11 landmarks were digitally marked by the operator. The landmarks are outlined in Table 1 and Figure 1.

### Facial measurements

**Figure 2:**
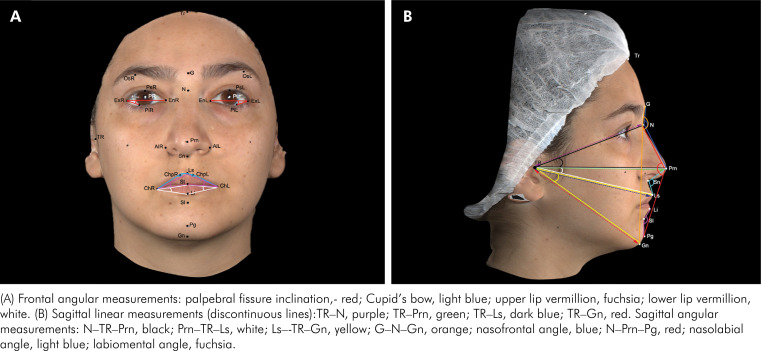
Frontal and sagittal view - Angular/Linear Measurements: Images obtained using Vectra Analysis Module Software (VAM elaboration, Canfield Scientific Inc.).

Linear measurements (Table 2) (Figures 1 and 2) and angular measurements (Table 3) were obtained.

**Table 2:** Comparative analysis of linear measures (mm) between women in groups G1 (20 –40 years) and G2 (41–65 years)

		G1 (n = 37)	G2 (n = 47)	
Linear measures (Total = 23) (mm)		Mean ± SD	Mean ± SD	p-value
		(IQR: 25^th^; 50^th^; 75^th^)	(IQR: 25^th^; 50^th^; 75^th^)	
Frontal view				
Total facial height measurement				
	Total facial height [Tr–Gn]	176.53 ± 8.72	176.96 ± 9.72	0.834
Upper-third measurement				
	Upper third height [Tr–G]	53.17 ± 6.48	53.49 ± 8.12	0.844
Middle-third measurement				
	Middle-third height [G–Sn]	61.62 ± 4.63	61.36 ± 4.81	0.802
	Palpebral fissure width [Mean Ex(R/L)–En(R/L)]	30.86 ± 1.74	29.24 ± 1.49	< 0.001**
	Palpebral fissure height [Mean Ps(R/L)–Pi(R/L)]	16.84 ± 1.95	15.52 ± 2.16	0.005
	Intercanthal width [En(R)–En(L)]	31.38 ± 2.79	32.38 ± 2.68	0.098
	Interpupillary width [P(R) - P(L)]	60.48 ± 3.18	61.69 ± 3.29	0.094
	Binocular width [Ex(R) - Ex(L)]	89.05±10.81 (88.38; 91,29; 93.96)	89.14±4.12 (86.05; 89.21; 91.57)	< 0.001***
	Upper eyelid height [Mean Os(R/L)–Ps(R/L)]	17.09 ± 4.12	15.13 ± 3.50	0.021
	Nasal width [Al(R) - Al(L)]	31.24 ± 2.93	33.03 ± 3.27	0.011
	Nasal height [N–Sn]	49.76 ± 3.06	50.55 ± 3.54	0.284
Lower third measurements				
	Lower-third height [Sn–Gn]	66.28 ± 4.52	66.48 ± 5.04	0.850
	Lip philtrum height [Sn–Ls]	15.58 ± 3.05 (13.50; 14.65; 17.61)	17.66 ± 8.01 (14.40; 16.53; 18.53)	0.045
	Lip philtrum width [Cph(R)-Cph(L)]	11.56 ± 2.11	11.55 ± 2.01	0.992
	Upper lip height [Sn–St]	21.85 ± 2.65	21.92 ± 2.63	0.900
	Upper lip vermilion height [Ls–St]	7.58 ± 1.32 (6.46; 8.01; 8.59)	6.28 ± 1.76 (5.36; 6.20; 7.06)	< 0.001***
	Lower lip vermilion height [St-Li]	10.08 ± 1.46	8.07 ± 1.81	< 0.001**
	Lower lip height [St-Sl]	17.60 ± 2.42	16.90 ± 2.56	0.205
	Mouth width [Ch(R)–Ch(L)]	48.91 ± 6.42 (47.94; 50.12; 52.20)	50.74 ± 6.75 (48.82; 51.52; 54.03)	0.021
Sagittal view				
	T(R)–N	116.26 ± 5.30	118.80 ± 4.71	0.022
	T(R)–Prn	131.17 ± 5.99	134.49 ± 5.23	0.008
	T(R)–Ls	125.08 ± 4.80	127.02 ± 4.92	0.073
	T(R)–Gn	136.78 ± 5.38	138.97 ± 5.40	0.068

Abbreviations: SD: Standard deviation; IQR: Interquartile range; R: Right; L: Left;

* statistically significant differences (p < 0.05);

** t-test;

*** Mann-Whitney test. Bonferroni correction.

**Table 3:** Comparative analysis of angular measurements (degrees) between women (n= 84) in groups G1 (age range: 20–40 years) and G2 (age range: 41–65 years)

		G1 (n = 37)	G2 (n = 47)	
Angular measures (Total = 12) (°)		Mean ± SD	Mean ± SD	p-value
		(IQR: 25^th^; 50^th^; 75^th^)	(IQR: 25^th^; 50^th^; 75^th^)	
Frontal view				
	Palpebral fissure inclination [Mean Ex(R/L)–En(R/L)–Pi(R/L)]	19.37 ± 3.28	17.79 ± 3.24	0.030
	Upper lip vermillion [Mean Ls–Ch(R/L) St]	13.40 ± 1.96 (12.37; 13.54; 14.38)	10.46 ± 2.75 (8.94; 10.49; 11.55)	< 0.001***
	Lower lip vermillion [Mean Li–Ch(R/L)–St]	19.99 ± 3.22	15.09 ± 3.32	< 0.001**
	Cupid's bow [Mean Ch(R/L)–Chp(R/L) –Ls]	129.13 ± 5.67	128.27 ± 5.28	0.472
Sagittal view				
	N–T(R)–Prn	19.62 ± 3.24 (18.05; 19.04; 19.96)	21.70 ± 17.26 (17.91; 19.26; 20.25)	0.648
	Prn - T(R) – Ls	13.94 ± 4.30 (12.11; 13.09; 14.52)	13.73 ± 1.52 (12.55; 14.00; 14.71)	0.218
	Ls–T(R)–Gn	22.83 ± 3.22 (21.57;22.32;23.67)	21.52 ± 2.12 (20.20; 21.49; 22.49)	0.014
Nasofrontal angle				
	[G–N–Prn]	143.59 ± 5.71 (141.13; 144.88; 147.21)	145.12 ± 19.65 (142.73; 146.27; 149.83)	0.037
	Pg–N–Prn	30.42 ± 3.24	29.95 ± 3.35	0.520
Nasomental angle				
	[N-Prn-Pg]	130.73 ± 4.00	130.82±4.43	0.923
Nasolabial angle				
	[Prn-Sn-Ls]	126.86 ± 8.29	123.45 ± 8.16	0.063
Labiomental angle				
	[Li-Sl-Pg]	137.74 ± 12.51	140.82 ± 9.60	0.205

Abbreviations: SD: Standard deviation; IQR: Interquartile range; R: Right; L: Left;

* statistically significant differences (p < 0.05);

** t-test;

*** Mann-Whitney test. Bonferroni correction.

### Data analysis

The data were analyzed using JAMOVI software (Jamovi v.1.2; Jamovi project). All variables were assessed using the Shapiro-Wilk test to assess normality. The t-test was applied for parametric results and the Mann–Whitney test for non-parametric results to determine the significance of linear and angle measurements between groups. The Bonferroni correction was applied to adjust for multiple t-tests, setting a new significant threshold at p < 0.001. The results are presented as mean, standard deviation, or median/interquartile range.

## Results

This study included 84 Caucasian women divided into two groups: G1, comprising 37 women aged 20–40 years (mean age 28.38 ± 7.02 years; p ≤ 0.001) and G2, comprising 47 women aged 41–65 years (mean age 52.26 ± 6.63 years; p = 0.097). An independent t-test was applied between groups for the variable age, revealing a statistically significant difference (p < 0.001). A total of 35 facial measurements (23 linear and 12 angular) were performed.

### Comparative analysis of linear measurements (mm) and angular measurements (degrees)

Statistically significant differences were observed in 4 out of 23 linear measurements and 2 out of 12 angular measurements (Tables 2 and 3).

## Discussion

This study evaluated facial variations in the linear and angular measurements of 84 adult Caucasian Brazilian women of different age groups using a stereophotogrammetry system. The results revealed significant differences in 4 out of 23 linear measurements and 2 out of 12 angular measurements.

The width of the palpebral fissure and binocular width in the periorbital region were higher in G1 than in G2, corroborating the findings of Kwon et al.,^1^ who observed similar morphological changes in the periorbital region in a sample of 192 Korean women categorized into three age groups (20–39, 40–59, and 60–79 years). Windhager et al.^19^ used a 3D scanning system to analyze the facial images of 56 Croatian women aged 26–89 years. The identified patterns of facial aging in pre-menopausal (aged 40–45 years) and post-menopausal (aged 55–65 years) women, with morphological variations comparable to our results in G2. These changes included a reduction in fissure height and ptosis of the upper and lower eyelids in the orbital region, leading to an impression of rounder and smaller eyes. Additionally, Bosch, Leenders, and Mulder^20^ investigated 320 individuals (age range: 10–89 years) and observed that the palpebral fissure width decreased gradually by 2.5 mm from the average age of 35 to 85 years, reaching approximately 23.5 mm±2.1 mm in women with a mean age of 85 years. This reduction in palpebral fissure width may be attributed to the laxity of the lateral and middle canthal structures.^21,22^ This finding was consistent with our results, which indicated that the width of the palpebral fissure was lower in G2 (29.24 ± 1.49 mm) than in G1 (30.86 ± 1.74 mm), with a statistically significant difference between the groups. However, the maximum age of participants included in our study was 65 years.

Our findings demonstrate that linear measurements of the lips in Brazilian Caucasian women exhibit changes during the aging process. These changes include shortening of the red portions of both the upper (G1: 7.58 ± 1.32; G2: 6.28 ± 1.76) and lower lips (G1: 10.08 ± 1.46; G2: 8.07 ± 1.81). These results were consistent with those reported by Chong et al.^22^ in their study of age-related changes in lip morphology in Chinese women aged 20–60 years. The current research findings illustrate a noticeable decline in the angles of both upper and lower lip vermilion as individuals age. This trend mirrors the widely accepted notion that lips tend to appear sunken over time owing to the deterioration and depletion of collagen and elastin fibers in the surrounding tissues.^23^ Additionally, Iblher, Stark, and Penna^24^ observed that aging prompts lip redistribution owing to collagen and elastin fiber degeneration, skin thinning, and changes in the orbicularis oris muscle. Consequently, the loss of structural support leads to lip ptosis and sagging of the labial tubercle,^1,24^ ultimately resulting in a decrease in the angle of the vermilion border.^23^

The substantial facial changes observed in G2 may be associated with the age of menopausal onset in Brazilian women.^25^ This age-related transition is characterized by a significant decline in estrogen production, contributing to skin aging, reduced skin thickness, and other degenerative alterations in dermal elastic tissue, primarily due to the depletion of underlying collagen.^19,26,27^ However, the primary objective of this study was to investigate the variations in facial morphology among Brazilian women. Consequently, we did not examine facial aging changes or their potential associations with hormonal and environmental influences. This limitation emphasizes the need for additional research to thoroughly explore how these factors interact with facial aging in Caucasian Brazilian women. A major limitation of a cross-sectional study is the scarcity of studies involving mixed populations, such as Caucasian Brazilians, for comparison in the literature. Furthermore, longitudinal studies are imperative for establishing parameters and comparing results.

## Conclusions

The most relevant changes in facial features were observed in women aged 20–65 years, indicating a decrease in palpebral fissure width, shorter upper and lower lip vermilion, and a reduction in upper and lower vermilion angles. When examining the facial thirds, the upper third remained unaffected, the middle third displayed changes in palpebral and binocular measures, and the lower third showed decreased upper and lower lip vermilion heights. Our findings may provide potential insights for clinicians in developing facial rejuvenation procedures as well as for forensic purposes and surgical planning.
